# Curability of Consumption Considered

**Published:** 1851-03

**Authors:** 


### ARTICLE IV.

*The curability of Consumption, considered in reference to a new method of ascertaining the healthy or diseased condition of the Lungs, &c.* By M. MATTSON, M. D., Fellow of the Massachusetts Medical Society &c., Boston. (From the author.)

This is a pamphlet of 16 pages, reprinted from the Boston Medical and Surgical Journal. It treats of the nature and uses of the *Spirometer* as a means of detecting diseases of the lungs; giving the laws of its use proposed by Dr. Hutchinson of London, who is the discoverer of some of the relations of the capacity of the lungs to disease, and the inventor of this instrument for measuring that capacity, by measuring the air expired. It also treats briely of the principles of cure of consumption, referring to the ætiology and pathology of the disease. It is a very interesting paper. We shall refer to the subject again on another page, by quoting Prof. Jackson's clinique.

